# Pharmacokinetic assessment of tacrolimus in combination with deoxyschizandrin in rats

**DOI:** 10.3389/fphar.2024.1344369

**Published:** 2024-05-23

**Authors:** Jianguo Sun, Zhipeng Wang, Na Liu, Zhijun Liu, Lili Cui, Xia Tao, Wansheng Chen, Shouhong Gao, Zhijun Wu

**Affiliations:** ^1^ Department of Pharmacy, Second Affiliated Hospital of Naval Medical University, Shanghai, China; ^2^ College of Traditional Chinese Medicine, Yunnan University of Traditional Chinese Medicine, Kunming, China; ^3^ The Fourth Retired Veteran Cadre’s Sanatorium of Fengtai District, Beijing, China

**Keywords:** tacrolimus, deoxyschizandrin, pharmacokinetic, UHPLC-MS/MS, rats

## Abstract

**Background:**

Tacrolimus (Tac) is commonly used for postoperative immunosuppressive therapy in transplant patients. However, problems, for example, low bioavailability and unstable plasma concentration, persist for a long time, Studies have reported that the deoxyschizandrin could effectively improve these problems, but the pharmacokinetic parameters (PKs) of Tac combined with deoxyschizandrin are still unknown.

**Method:**

In this study, an UHPLC-MS/MS method has been established for simultaneous quantitation of Tac and deoxyschizandrin. The PKs of Tac influenced by different doses of deoxyschizandrin after single and multiple administrations were analyzed, and the different impact of deoxyschizandrin and Wuzhi capsule on PKs of Tac were compared.

**Result:**

The modified UHPLC-MS/MS method could rapid quantification of Tac and deoxyschizandrin within 2 min using bifendatatum as the internal standard (IS). All items were successfully validated. The *C*_max_ of deoxyschizandrin increased from 148.27 ± 23.20 to 229.13 ± 54.77 ng/mL in rats after multiple administrations for 12 days. After co-administration of 150 mg/mL deoxyschizandrin, Tac had an earlier *T*_max_ and greater *C*_max_ and AUC_0–t_, and the *C*_max_ and AUC_0–t_ of Tac increased from 14.26 ± 4.73 to 54.48 ± 14.37 ng/mL and from 95.10 ± 32.61 to 315.23 ± 92.22 h/ng/mL, respectively; this relationship was positively proportional to the dosage of deoxyschizandrin. In addition, compared with Wuzhi capsule, the same dose of deoxyschizandrin has a better effective on Tac along with more stable overall PKs.

**Conclusion:**

An UHPLC-MS/MS method was established and validated for simultaneous detection of deoxyschizandrin and Tac. Deoxyschizandrin could improve the *in vivo* exposure level and stability of Tac, besides, this effect is better than Wuzhi capsule in same dose.

## 1 Introduction

Tacrolimus (Tac, or FK506), an immunosuppressor, is the first-line medication for organ transplantation, such as kidney and liver (Yu et al., 2018), which is used alone or in combination with other drugs to prevent acute rejection and improve long-term graft survival after transplantation (Bowman and Brennan, 2008; Coste and Lemaitre, 2022). Nevertheless, the therapeutic index of Tac is very narrow in long-term regimen, and the average bioavailability of Tac is merely 25% (Abushammala, 2021), as a result, it is often difficult to reach and/or maintain the effective plasma concentration of Tac, and it varies dramatically among individuals, too (Staatz and Tett, 2004). Due to the low therapeutic index and capricious pharmacokinetics, the possibility of treatment failure and toxicity increased (Paglialunga et al., 2017). Currently, therapeutic drug monitoring (TDM) is standard clinical method to monitor the plasma level of Tac in transplant patient, but the TDM still has some limitations, such as complex detection methods and lagged detection results (Brunet et al., 2019). There is strong association of hepatotoxicity with abnormal elevated plasma level of Tac, inducing even fatal hepatic necrosis (Banas et al., 2020). Therefore, it is extremely unsafe to simply increase the dose of Tac in order to reach an effective plasma concentration. It is necessary to find a safe and economical method that can maintain the plasma concentration and increase the therapeutic index of Tac.

Growing researches confirmed that traditional Chinese medicine and phytotherapy in combination with drugs showed great improvement (Li et al., 2022), for instance, Wuzhi capsule combined with Tac and tetrandrine combined with corticosteroids (Wang et al., 2018; Cheng et al., 2021). These herb-drug combinations may solve many problems, including reducing toxicity and resistance, increasing bioavailability, maintaining plasma level of drug, etc. (Wang et al., 2014). In the past few years, the traditional Chinese medicine Wuzhi capsule prescribed with Tac has been widely applied in China (Zhang et al., 2018; Teng et al., 2022), and our group found that it could significantly increase the effective plasma concentration of Tac (Wei et al., 2010), and numerous studies have reported that the components in Wuzhi capsule can inhibit the activity of P-gp and CYP3A (Iwata et al., 2004; Huyke et al., 2007), especially the deoxyschizandrin, which may maintain the plasma concentration and increase the bioavailability of Tac through inhibiting the P-gp-mediated efflux and CYP3A-mediated catabolism of Tac (Wan et al., 2010; Qin et al., 2014b). Although the combination of Wuzhi capsule and Tac is already commonly used in China (Jing et al., 2021), the complex compositions of Wuzhi capsule make it difficult to control the quality across different manufacturers and batches (Qin et al., 2014a), which hindered its clinical application; lastly, there is currently no clinically applied compound preparation of Tac. Deoxyschizandrin is the most active ingredient in Wuzhi capsule, and some studies have confirmed its potential to improve the bioavailability of Tac (Qu et al., 2022). However, the PKs of deoxyschizandrin in combination with Tac remain unclear. Therefore, it is crucial to elucidate the influence of deoxyschizandrin, but not the Wuzhi capsule, on the PKs of Tac, which may be more potential to improve the therapeutic index of Tac and to develop a Tac-based compound preparation.

In this study, an UHPLC-MS/MS method was established for rapid, accurate, and simultaneous determination of Tac and deoxyschizandrin in rat plasma. This method reduced the analysis time from 3.5 min to 2 min, and replaced the liquid-liquid extraction method with a simple and economical protein precipitation method (Wei et al., 2014). Besides, the influence of single and multiple doses of deoxyschizandrin on the PKs of Tac were assessed, and the results may provide reference for safe application of Wuzhi capsule combined with Tac in clinic and the development of compound preparation of Tac and deoxyschizandrin.

## 2 Materials and methods

### 2.1 Chemicals and regents

Standards of Tac (Lot: S31618) (purity>98%), deoxyschizandrin (Lot:20210612) (purity>98%), and bifendatatum (Lot: Y06O7C22268) (internal standard, IS) (purity>98%) were all provided by Shanghai Yuanye Bio-Technology CO., Ltd (Shanghai, China), and the Wuzhi capsule was purchased from Sichuan Hezheng Pharmaceutical Co., Ltd. Acetonitrile and methanol in mass spectrometry grade were purchased from Merck Company (Kenilworth, United States of America). Formic acid in analytical grade was obtained from Tedia Company (Fairfield, United States of America). HPLC-grade isopropanol was obtained from Shanghai Titan Technology CO., Ltd. (Shanghai, China), and distilled water was supplied by Watsons Distilled Water CO., Ltd. (Shenzhen, China).

### 2.2 Instrumentation

All analyses were performed with a model 1,290 series UHPLC system equipped with an Agilent G1969-80230 online degasser, a G4220A binary pump, a G4226A autosampler, and a G1316C column oven, which was interfaced with an Agilent G6460A triple-quadrupole mass spectrometer and the mass spectrometer was equipped with electrospray ionization source (Agilent Technologies, Santa Clara, United States of America). All raw data were acquired and analyzed using Agilent Mass Hunter data processing software (version B.06.00). A VX230 vortex-mixer was purchased from Labnet (Edison, New Jersey, United States of America); a 5810R low temperature high speed centrifuge and single lane pipette are products of Eppendorf Company (Eppendorf, Hamburg, Germany). The SK7200H ultrasound instrument is provided by Shanghai Kedao Ultrasonic Instrument Co., Ltd (Shanghai, China). The standards were weighed using an electronic balance provided by Sartorius Company (CPA225D 1/ppm, Gottingen, Germany).

### 2.3 LC-MS/MS conditions

The chromatographic separation of two analytes was achieved in an Agilent ZORBAX SB-C18 column (2.1 mm * 100 mm, 3.5 μm, Agilent, MA, United States of America) at a flow rate of 0.3 mL/min with column temperature set at 35°C. The mobile phase A was acetonitrile, and mobile phase B was 0.1% formic acid in water, and the initial mobile phase of gradient elution program was consisted of 60% phase A and 40% phase B. The sample injection volume was set at 10 μL. Gradient elution program was supplied in Supplementary Table S1.

**FIGURE 1:**
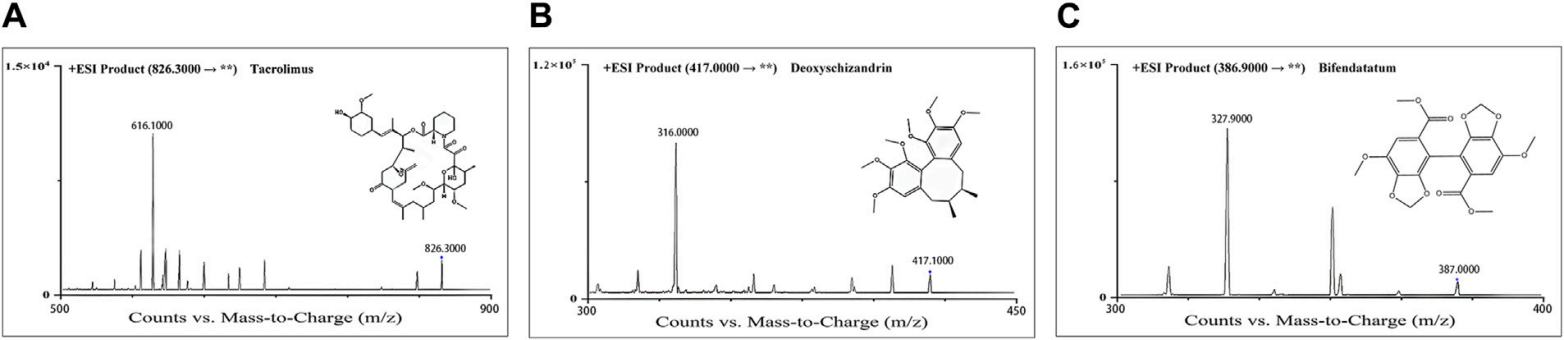
Product-ion chromatograms and chemical structures of Tac **(A)**, deoxyschizandrin **(B)**, and bifendatatum (IS) **(C)**.

The mass spectrometry detection was achieved on an Agilent 6460A mass spectrometer with ESI source. Data acquisition was operated in multiple reaction monitoring (MRM) mode, and the source temperature was 105°C. The settings of the mass spectrometer source were as follows: capillary voltage 4,000 V, sheath gas flow 12 L/min, temperature 400°C, dry gas (nitrogen) flow 10 L/min and temperature 350°C, nebulizer pressure 45 psi. All analytes were detected in positive ionization mode. The optimized MRM parameters are shown in Table 1. Collision gas (high purity liquid nitrogen) was set at 0.25 MPa. The structures of both two analytes and IS are display in Figure 1.

**TABLE 1:** Optimized mass spectrometry parameters of two analytes and IS.

Analyte	Precursor ion(m/z)	Product ion(m/z)	Fragmentor (V)	Collisionenergy(eV)	Ionization mode
Tacrolimus	826.3	616.2	240	45	positive
Deoxyschizandrin	417.1	316.0	155	22	positive
Bifendatatum	386.9	327.9	140	16	positive

### 2.4 Preparation of standards and quality-control samples

To prepare the stock solution, approximately 2.00 mg of Tac, deoxyschizandrin and IS were accurately weighed and dissolved in methanol, respectively. The stock solutions of Tac and deoxyschizandrin with a final concentration of 1.00 mg/mL was obtained, and the IS stock solution was diluted to 100.00 ng/mL using methanol before use. The stock solutions were aliquoted and stored at −80°C. Combined working solutions were prepared by diluting the stock solution using 10% aqueous methanol at the following concentrations: 5.00, 10.00, 20.00, 50.00, 100.00, 200.00, 500.00, 1,000.00, and 2,000.00 ng/mL. Blank rat plasma was used to prepare the calibration standards. Quality-control (QC) samples of two analytes were prepared separately in the same manner at three concentrations, namely, 10 (low concentration), 100 (middle), 1,000 ng/mL (high), and all QC samples were stored at −80°C.

### 2.5 Blood sample pretreatment

The pretreatment was performed for the plasma sample by one-step protein precipitation, Briefly, 100 μL sample was transferred into a 1.5 mL EP tube prior to adding with 300 μL methanol(containing 200 ng/mL IS). The mixture was vortex-mixed for 30 s before centrifugation at 17,400 *g* for 10 min at room temperature, and 100 μL of the supernatant was diluted with 100 μL of acetonitrile (v/v, 1:1) Then, a 10 μL aliquoted of diluted solution was injected into the UHPLC-MS/MS system for analysis.

### 2.6 LC-MS/MS method validation

The methodology validation was completed according to FDA guidelines and Chinese Pharmacopoeia (2015 edition), and the linearity, inter- and intra-precision and accuracy, matrix effect and extraction recovery, stability and specificity of this method were validated according to previous study (Lu et al., 2022; Wang et al., 2022; Shu et al., 2016).

### 2.7 Animal experiment and sample collection

The protocol of the animal study was approved by the Experimental Animal Ethics Committee of the Shanghai University of Traditional Chinese Medicine. Specific Pathogen Free (SPF) grade SD rats, male, 8 weeks old, 200–250 g, were obtained from the animal experiment center of Shanghai University of Traditional Chinese Medicine. After passing quarantine, the rats were adaptive fed for 2 days. Rats were fasted for 12–14 h, followed by the initiation of pharmacokinetic study of Tac and deoxyschizandrin. This animal study was carried out in accordance with the National Institutes of Health Guide for the Care and Use of Laboratory animals.

In this study, 42 rats were divided equally into seven groups of six rats each, numbered NG2201-NG2207. Among them, the NG2201 and NG2202 group was dosed with 150 mg/kg deoxyschizandrin alone to study the PK of deoxyschizandrin in rats after single and multiple doses; the NG2203 group was administrate dosed with 1.0 mg/kg of Tac; the NG2204-NG2206 groups were dosed with 75, 150, and 300 mg/kg of deoxyschizandrin plus 1.0 mg/kg Tac, respectively, and the NG2207 group was dosed with the Wuzhi capsule (approximately equal to 150 mg/kg of deoxyschizandrin) plus 1.0 mg/kg of Tac (Teng et al., 2022). The detailed dosing regimen is shown in Table 2, and all dose in this experiment were calculated according to clinical dose. Blood samples (approximately 300 μL for each) for PK analysis were collected into lithium–heparin tubes. The plasma sampling schedules are outlined below: Blood samples were drawn pre-dose, at 5, 10, 20 and 30 min, and at 1, 1.5, 2, 4, 8 and 24 h after drug administration. The blood samples were centrifuged at 5,000 *g* for 15 min at 4°C to collect plasma samples, which were placed −80 refrigerator.

**TABLE 2:** Plans of rat dose and blood collection.

Group	Type of drug	Dose of drug	Blood collection time
NG2201	Deoxyschizandrin	150 mg/kg	D1-D2
NG2202	Deoxyschizandrin	150 mg/kg	D11-D12
NG2203	Tacrolimus	1 mg/kg	D1-D2
NG2204	Deoxyschizandrin plus tacrolimus	75 mg/kg and 1 mg/kg	D1-D2
NG2205	Deoxyschizandrin plus tacrolimus	150 mg/kg and 1 mg/kg	D1-D2
NG2206	Deoxyschizandrin plus tacrolimus	300 mg/kg and 1 mg/kg	D1-D2
NG2207	Wuzhi capsule plus tacrolimus	150 mg/kg and 1 mg/kg	D1-D2

### 2.8 Pharmacokinetics analysis

The rat plasma collected from each group was pretreated according to the method in section 2.5, and exposure levels of Tac and deoxyschizandrin were determined using the above established UHPLC-MS/MS method and the PKs were calculated using DAS software using non-compartment model (version 2.0, China Pharmacological Society). The data were presented as mean ± SD.

## 3 Results

### 3.1 Optimization of LC-MS/MS

Specifically. The ionization mode tested in positive and negative ionization modes showed that no precursor ions were found in the negative ionization mode, whereas the [M + H]+peak of the analytes were observed in the positive ionization mode with high responses. In addition, several common columns were tested, such as Agilent ZORBAX SB-C18 (2.1 × 100 mm, 3.5 µm), Agilent ZORBAX SB-C8 (2.1 × 150 mm, 5 µm), and Waters XBridge^®^ C18 (2.1 × 100 mm, 3.5 µm) and so on, and finally the ZORBAX SB-C18 column (2.1 mm × 100 mm, 3.5 μm) was selected to be the most appropriate for two analytes and IS separation. By comparing the common mobile phases, such as methanol, acetonitrile, and their mixture and others, the best mobile phase was determined to be acetonitrile and 0.1% formic acid in water. Besides, we tried both isocratic and gradient elution programs, and the retention times of two analytes and IS were 3.5 min by isocratic elution with 60% acetonitrile, while the retention times of analytes were reduced to less than 2 min with gradient elution with acetonitrile. The acetonitrile and 0.1% formic acid in water delivered in gradient program were the best mobile phases for the Tac, deoxyschizandrin, and IS. Previous studies have established UHPLC-MS/MS methods, which contain deoxyschizandrin and Tac (Wei et al., 2013), but the methods were time-consuming and inefficient, with complex pre-treatment methods for plasma samples (Wei et al., 2010). In this study, we improved the UHPLC-MS/MS method based on previously reported, and established a modified method that can be used for rapid and accurate detection of deoxyschizandrin and Tac. The mobile phases in this study were suitable for the separation and ionization of deoxyschizandrin, Tac and IS, as it provided good peak shapes and resolutions.

### 3.2 Sample pretreatment

Plasma is a complex matrix containing a variety of compounds, including proteins, lipids, soluble inorganic salts, and small polar molecules, etc., and these compounds can cause ion interference in LC-MS/MS analyses (Peng et al., 2016). Thus, pretreatment of plasma samples is an effective way to reduce the matrix effect, remove potential interferences, and improve the resolution and sensitivity of mass spectrometry detection (Hyötyläinen, 2009). The protein precipitation method was tested with methanol and acetonitrile with consideration of simplicity and cost. For this purpose, plasma samples were mixed with methanol (1:3, V/V), and the recovery of Tac reached about 92.36% and matrix effect of Tac was approximately 99.23%, the recovery of deoxyschizandrin reached 104.03% and matrix effect of deoxyschizandrin was approximately 106.49%, showing advantages compared to acetonitrile as precipitant. In addition, the dilution of supernatant with different solvents, for example, methanol, acetonitrile and initial mobile phase in different ratios were further assessed, and the 100 μL acetonitrile added into 100 μL supernatant brought better and more stable effect, and the matrix effects of both Tac and deoxyschizandrin were optimized to 99.23% and 106.49%.

### 3.3 Method validation

#### 3.3.1 Specificity

**FIGURE 2:**
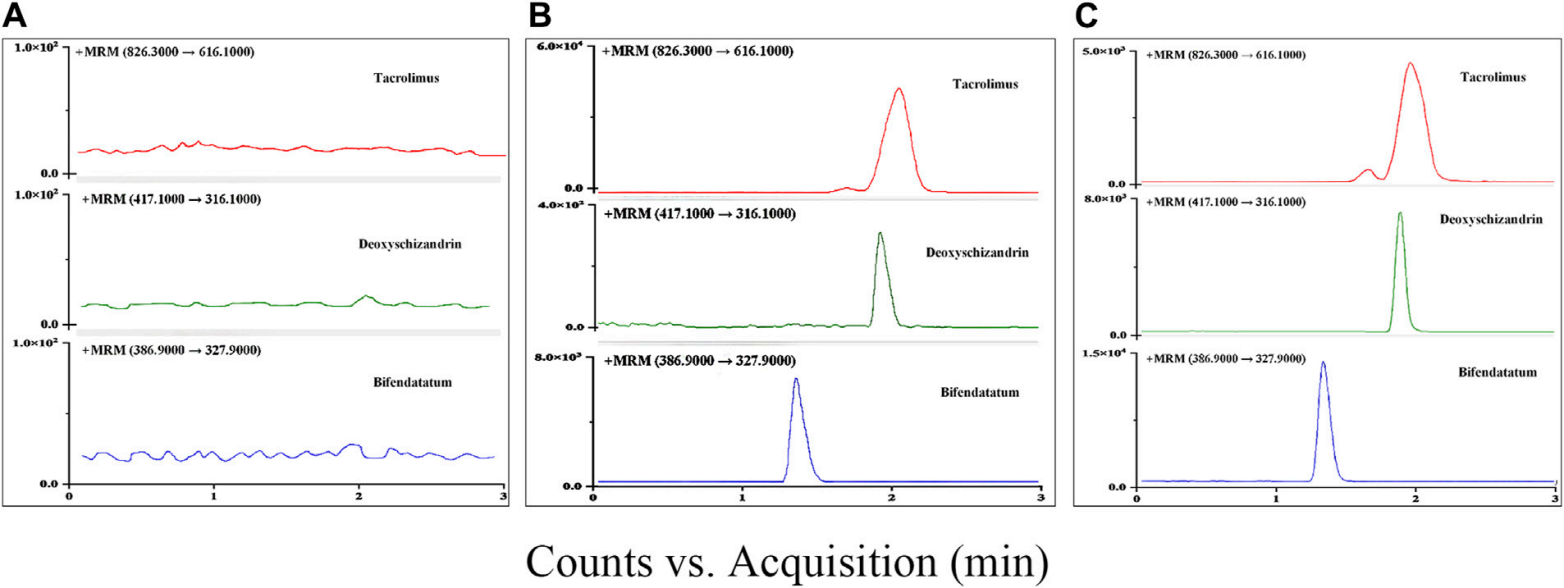
Typical chromatograms in MRM mode for Tac, deoxyschizandrin, and bifendatatum (IS): **(A)** Blank rat plasm. **(B)** LLOQ (Blank rat plasm add with 5 ng/mL of two analytes). **(C)** Blank rat plasm spiked with 1,000 ng/mL of two analytes.

The retention time of Tac and deoxyschizandrin was 2.0 min and 1.9 min, respectively, and a complete separation with endogenous compounds was achieved. The specificity assessment was completed through comparing the responses of analytes in blank samples, spiked samples and real samples, and the results showed that there were no interferences in the retention time of Tac, deoxyschizandrin and IS (Figure 2).

#### 3.3.2 Linearity and LLOQ

Nine calibration standards were prepared and the calibration curves were constructed for Tac and deoxyschizandrin under the weighing factor x^−1^; the linear range of the two analytes was between 5 and 2000 ng/mL, and the R2 were both greater than 0.99 (Table 3). The LLOQ was 5 ng/mL both for Tac and deoxyschizandrin. The deviations of back-calculation for all calibration standards were within ±15%.

**TABLE 3:** Linear regression equation, range, and correlation coefficients of two analytes.

Analyte	Calibration range (ng/mL)	Regression equations	*R* ^2^
Tacrolimus	5–2000	Y = 0.01547*X-0.9293	0.9933
Deoxyschizandrin	5–2000	Y = 0.00621*X-0.2537	0.9918

#### 3.3.3 Inter- and intra-day precision and accuracy

In this experiment, the intra-day and inter-day precision and accuracy were assessed at Low, Mid and High concentration levels in five replicates (n = 5). The results showed that the inter- and intra-day accuracy were within 1.76% and 2.28%, and RSD% values were less than 2.65%, which met the requirements (Supplementary Table S2).

#### 3.3.4 Matrix effect and recovery

The extraction recovery of the Tac and deoxyschizandrin were 92.36%–99.23%, 104.03%–107.93% respectively, and the matrix effect of the Tac and deoxyschizandrin were 99.23%–131.61% and 106.49%–144.34% respectively, and the RSD% of them were all less than 15% (Büttner et al., 2016) (Table 4).

**TABLE 4:** Extraction recovery and matrix effect data of the two analytes in rat blood (n = 6).

Analyte	Nominal Conc. (ng/mL)	Extraction recovery		Matrix effect	
		Mean % ± SD	RSD%	Mean % ± SD	RSD%
	10	99.23 ± 15.36	8.64	99.23 ± 10.37	13.81
Tacrolimus	100	94.78 ± 6.08		111.33 ± 9.05	
	1,000	92.36 ± 9.91		131.61 ± 7.30	
	10	106.75 ± 6.62	3.89	106.49 ± 8.78	14.20
Deoxyschizandrin	100	107.93 ± 1.51		132.13 ± 10.47	
	1,000	104.03 ± 4.65		144.34 ± 8.05	

#### 3.3.5 Stability

Two analytes in rat plasma at the three concentration levels (Low, Mid and High) were used to assessed stability, and for stability, two analytes were steady in room temperature for 8 h and in autosampler for 24 h, and the long-term stability and frozen-thaw stability also met the requirements The data proved that the deviations of analytes located within ±15% (Supplementary Table S3).

### 3.4 Application in pharmacokinetic study

An UHPLC-MS/MS method was established and validated for determining Tac and deoxyschizandrin, which was then applied to a pharmacokinetic study of them. A total of 420 plasma samples were collected from all rats at 10 time points according to the protocol. This method was applied to determine the contents of Tac and deoxyschizandrin in 420 plasma samples.

### 3.5 The PKs of deoxyschizandrin after single and multiple administrations

**FIGURE 3:**
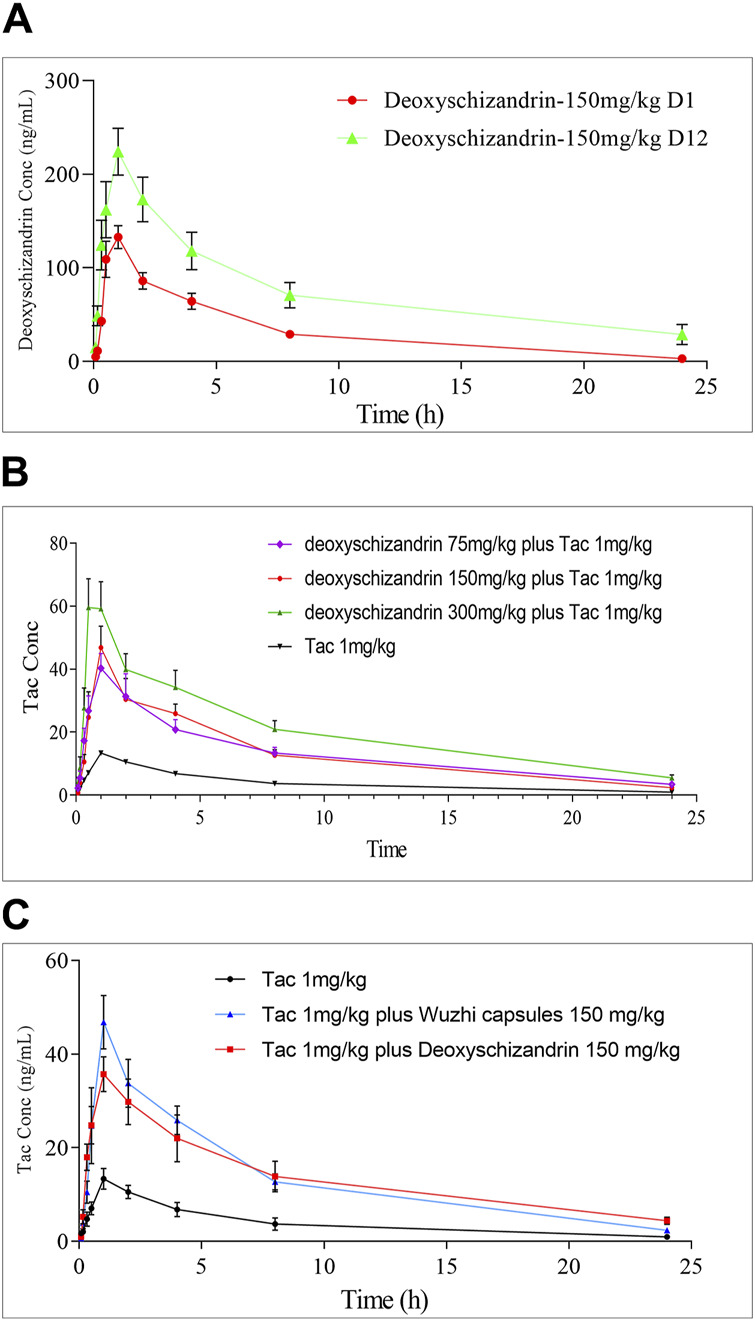
Time-concentration relationship of Tac, deoxyschizandrin and Wuzhi capsule. **(A)** Mean plasma concentration of deoxyschizandrin after 1 and 12 days of continuous dosing. **(B)** Mean plasma concentration of Tac after co-administration with 75, 150 and 300 mg/kg of deoxyschizandrin. **(C)** Mean plasma concentration of Tac after co-administration with the same dose of deoxyschizandrin and Wuzhi capsule.

NG2201 and NG2202 groups were designed to study the PKs of deoxyschizandrin after single and multiple dosing, in which group NG2201 was administered 150 mg/kg deoxyschizandrin and plasma samples were collected on the first day, and group NG2202 was collected after 12 days of continuous administration of 150 mg/kg deoxyschizandrin. No adverse reactions occurred in the rats, indicating the safety of long-term oral administration of deoxyschizandrin. In addition, pharmacokinetic results showed that the *C*_max_ of deoxyschizandrin in rats increased from 148.27 ± 23.20 to 229.13 ± 54.77 ng/mL after multiple administrations, and the AUC_0–t_ elevated from 785.77 ± 173.66 to 1806.48 ± 707.19 h/ng/mL (Table 5), and the other parameters such as AUC_0→∞_, MRT, and *T*_1/2_ were also significantly enhanced (Figure 3A). These results suggests that multiple administrations increased the concentration of deoxyschizandrin in rats, which may exert their pharmacological effects for a longer period of time and obtain better efficacy.

**TABLE 5:** PKs of deoxyschizandrin in rat aftersingle and multiple administrations.

Parameter	Groups	
	NG2201	NG2202
*T*_max_ (h)	0.92 ± 0.20	1.50 ± 1.23
*C*_max_ (ng/mL)	148.27 ± 23.20	229.13 ± 54.77
*T*_*1/2*_ (h)	4.48 ± 0.53	9.10 ± 7.83
AUC_0→t_ (ng. h/mL)	785.77 ± 173.66	1806.48 ± 707.19
AUC_0→∞_ (ng. h/mL)	806.40 ± 175.79	2413.16 ± 1716.77
AUMC_0→t_ (ng. h^2^/mL)	4,088.71 ± 1,056.37	13,360.23 ± 8,009.79
MRT (h)	5.17 ± 0.33	6.95 ± 1.42

^a^ NG2201: 150 mg/kg Deoxyschizandrin single; NG2201: 150 mg/kg deoxyschizandrin multiple.

### 3.6 The PKs of tac

The basic PKs of Tac in rats was calculated in NG2203 group, and plasma samples were gathered from rats after administration of Tac at a dose of 1 mg/kg. The analysis data showed that the *C*_max_ of Tac was 14.26 ± 4.73 ng/mL and varied widely between rats in the group; the AUC_0–t_ was 95.10 ± 32.61 h/ng/mL; the AUC_0→∞_ was 105.20 ± 31.28 h/ng/mL; the MRT was 6.54 h, and the *T*_1/2_ was 7.3 ± 3.06 h (Table 6).

**TABLE 6:** PKs of Tac in rat after co-administration with different doses of deoxyschizandrin or with Wuzhi capsule.

Parameter	Groups				
	NG2203	NG2204	NG2205	NG2206	NG2207
Tmax (h)	1.33 ± 0.52	1.17 ± 0.41	1.08 ± 0.49	0.75 ± 0.27	1.50 ± 0.55
Cmax (ng/mL)	14.26 ± 4.73	43.20 ± 13.72	54.48 ± 14.37	67.40 ± 18.64	43.25 ± 21.86
T1/2 (h)	7.30 ± 3.06	7.31 ± 1.04	5.04 ± 1.95	6.59 ± 1.62	8.27 ± 2.20
AUC0→t (ng. h/mL)	95.10 ± 32.61	313.58 ± 99.42	315.23 ± 92.22	486.27 ± 149.08	340.40 ± 193.26
AUC0→∞ (ng. h/mL)	105.20 ± 31.28	350.89 ± 117.19	331.24 ± 97.95	534.10 ± 176.32	388.49 ± 216.74
AUMC0→t (ng. h2/mL)	613.05 ± 182.15	2107.22 ± 701.25	1915.12 ± 621.91	3,315.05 ± 1,100.65	2395.69 ± 1,225.18
MRT (h)	6.54 ± 0.55	6.69 ± 0.25	6.02 ± 0.53	6.76 ± 0.31	7.23 ± 1.04

^a^ NG2203: 1.0 mg/kg of Tac; NG2204: 1.0 mg/kg of Tac plus 75 mg/kg deoxyschizandrin; NG2205: 1.0 mg/kg of Tac plus 150 mg/kg deoxyschizandrin; NG2206: 1.0 mg/kg of Tac plus 300 mg/kg deoxyschizandrin; NG2207: 1.0 mg/kg of Tac plus Wuzhi capsule.

### 3.7 The PKs of tac after combined administration of deoxyschizandrin

In this study, the influence of three different doses of deoxyschizandrin on the PKs of Tac were assessed. NG2204-NG2206 groups were administered 75, 150, and 300 mg/kg deoxyschizandrin plus 1 mg/kg Tac, respectively. Apparently, the *C*_max_, AUC_0–t_, and AUC_0→∞_ of Tac in rats significantly improved as the dose of deoxyschizandrin increased. For example, the *C*_max_ of Tac increased 3-4-fold and plasma concentrations were more stable after co-administered the deoxyschizandrin; the *T*_max_ of Tac was shortening along with increasing dose of deoxyschizandrin. Besides, the other PKs fluctuated in a small range, independent of the dose variation of deoxyschizandrin (Figure 3B). Taking group NG2205 as an example, after co-administrated with 150 mg/mL deoxyschizandrin, the *C*_max_ of Tac in rats increased from 14.26 ± 4.73 to 54.48 ± 14.37 ng/mL, and the AUC_0–t_ from 95.10 ± 32.61 to 315.23 ± 92.22 ng/mL (Table 6).

The results showed that the deoxyschizandrin could remarkably increase the plasma concentration of Tac, narrow the inter-individual concentration fluctation, and shorten *T*_max_ in rats. The combination of deoxyschizandrin with Tac may solve the problems of low bioavailability and unstable exposure of Tac.

### 3.8 The PKs of tac after combined with wuzhi capsule

To compare the influences of deoxyschizandrin and Wuzhi capsule on Tac, after administration of Wuzhi capsule (equivalent to 150 mg/kg deoxyschizandrin) and 1.0 mg/kg of Tac in rats, the plasma concentrations of Tac at different time points were measured and the PKs were calculated. The analysis data showed that the *C*_max_ of Tac was 43.25 ± 21.86 ng/mL and the AUC_0–t_ was 340.40 ± 193.26 h/ng/mL; the AUC_0→∞_ was 388.49 ± 216.74 h/ng/mL; the MRT was 7.23 ± 1.04 h, and the *T*_1/2_ was 8.27 ± 2.20 h (Table 6). Obviously, compared with administration of deoxyschizandrin, the *T*_max_ of Tac was later with administration of Wuzhi capsule, and although the mean *C*_max_ was only slightly lower by 11.23 ng/mL, the fluctuation of Tac concentration was significantly larger (Figure 3C). These results suggest that the PKs of Tac co-administered with deoxyschizandrin are more stable and effective.

## 4 Discussion

Numerous studies have reported that the combination of Wuzhi capsule with Tac could significantly improve the bioavailability and therapeutic index of Tac (Zhao et al., 2017; Seo et al., 2021), Although the application of this herb-drug combination in China has been very widespread (Wang et al., 2019), the quality of Wuzhi capsule is difficult to ensure due to different manufacturers and batches (Zhang et al., 2019; Peng et al., 2021), which often leads to unsteady exposure of Tac. There is still no a clinically available Tac-based compound preparation yet (Jing et al., 2021), thus, this study explored the combination of Tac and deoxyschizandrin in SD rats, and chose 10 time points for plasma collection referred to previous published reports (He et al., 2022). The doses of the Tac and deoxyschizandrin were determined referenced to our previous studies and the clinical dose (Wei et al., 2013).

We firstly administered deoxyschizandrin at a dose of 150 mg/kg and studied the changes in PKs in rats after a single dose and multiple doses. After consecutive 12 days of administration, the *C*_max_ of deoxyschizandrin in rat was increased by about 1.5-fold (from 148.27 ± 23.20 to 229.13 ± 54.77 ng/mL), and the *T*_max_, *T*_1/2_, AUC_0–t_, AUC_0→∞_, and the MRT significantly improved. The trends in these results were similar to previously published studies (Deng et al., 2008; Qin et al., 2014b), which indicated higher plasma concentration and slower metabolism of deoxyschizandrin in rats after multiple consecutive administrations, and no adverse effects were demonstrated during the experiment, too. Li WL, et al. reported that administration of deoxyschizandrin for consective 3 day could inhibit the catabolism of midazolam, which is a specific CYP3A substrate (Li et al., 2012), thus the activity of CYP 3A might be suppressed by the deoxyschizandrin; and another study showed that the CYP2C19 could be induced by the deoxyschizandrin and other components in Wuzhi capsule, such as schisandrin B (Yuan et al., 2020), but this study did not measure these metabolic enzymes. Then, this study co-administrated Tac and deoxyschizandrin in rat and firstly reported the PKs changes of the *C*_max_ and AUC_0–t_ obtained for Tac increased from 14.26 ± 4.74 ng/mL to 54.48 ± 14.37 ng/mL and from 95.10 ± 32.61 h/ng/mL to 315.23 ± 92.22 h/ng/mL after combined with 150 mg/mL deoxyschizandrin, respectively, and this effect was positively correlated to the dose of deoxyschizandrin. These results showed that the absorption of Tac *in vivo* was limited, as the T_max_ decreased along with the increase of deoxyschizandrin dose. The plasma concentration was very unstable, but this awkward situation was improved after co-administration of deoxyschizandrin (coefficient of variations of PKs decreased of Tac). Deoxyschizandrin was proved to inhibit CYP3A4 and 3A5 in a time-dependent manner but reversibly inhibited CYP3A5, which was considered to increase the exposure level of Tac (He et al., 2021). This study finally compared the deoxyschizandrin and Wuzhi capsule in combination with Tac at the same dose, and the result showed that co-administration of deoxyschizandrin with Tac had higher *C*_max_ and more stable other PKs (smaller coefficient of variations of PKs of Tac when combined with deoxyschizandrin).The inhibition of deoxyschizandrin to CYP3A4 and 3A5 was a time-dependent manner, but the application of Wuzhi capsule brought shorter MRT time of deoxyschizandrin (Wei et al., 2010; Wu et al., 2014), which may weaken this inhibition of CYP3A. All these results suggested that deoxyschizandrin may be a better alternative to Wuzhi capsule.

The mechanism of herb-drug interaction between deoxyschizandrin and Tac has not been conclusively determined, and current studies only generally indicate that Wuzhi capsule could inhibited P-gp-mediated efflux (Qin et al., 2010a; Qin et al., 2010b) and CYP3A-mediated metabolism (Chen et al., 2020; Kou et al., 2022) of Tac, which resulted in an increased systemic exposure of Tac. These studies consider that Tac is a substrate of P-gp, and is mainly metabolized by cytochrome CYP3A in the liver and the small intestine (Picchianti-Diamanti et al., 2014; Krogstad et al., 2018; Prytuła et al., 2019; Edavalath et al., 2022); so, if drugs that affect the activity of P-gp or CYP3A may influence the pharmacokinetics of Tac (Wen et al., 2023). Previously published paper has shown that several constituents from Wuzhi capsule could inhibited activity of P-gp and CYP3A (Xue et al., 2013; Chen et al., 2022). And thus, the PKs of Tac could be influenced. So, the ways of deoxyschizandrin improving the PKs of Tac may function through the inhibition of P-gp-mediated efflux and CYP3A-mediated metabolism of Tac.

This study still has some shortcomings: 1) the expression or activity of these metabolic enzymes and/or transporters were not determined in the rats. These results may deepen the understanding of exposure level of Tac increased by deoxyschizandrin. 2) the other components in Wuzhi capsule have not been measured in this study as some studies have reported the other components could inhibit the CYP 3A enzyme, and may impact our results; 3) all our results necessitate further studies in human to verify if the deoxyschizandrin can and how increase the exposure level and narrow the concentration fluctuation of Tac.

## 5 Conclusion

In this study, an UHPLC-MS/MS method was established for the simultaneous detection of deoxyschizandrin and Tac, which showed simple and economical plasma sample preparation and short analysis time. This method has been successfully applied to the pharmacokinetic evaluation of Tac in rats after co-administration of deoxyschizandrin. This study firstly reported an increased PKs of Tac after combining with different doses of deoxyschizandrin. Moreover, deoxyschizandrin has a superior influence on Tac than that of Wuzhi capsule, with higher *C*_max_ and more steady PKs. These works may provide a basis for preparing Tac-based compound preparation with deoxyschizandrin and further mechanism researches and clinical studies are required to verify these results.

## Data Availability

The original contributions presented in the study are included in the article/Supplementary Material, further inquiries can be directed to the corresponding authors.
