# Supplementary material for: Pharmacokinetic assessment of tacrolimus in combination with deoxyschizandrin in rats
Source: Front Pharmacol. 2024 May 23;15:1344369. doi: 10.3389/fphar.2024.1344369 (PMC11188489; doi:10.3389/fphar.2024.1344369)
Supplement: Supplementary file 1 [file Table1.DOCX]

Table S1: Gradient elution program of two analytes and IS

| Time (min) | Mobile phase A (%) | Mobile phase B (%) | Flow velocity (ml/min) |
| --- | --- | --- | --- |
| 0 | 60 | 40 | 0.3 |
| 0.5 | 100 | 0 | 0.3 |
| 3 | 100 | 0 | 0.3 |

Table S2: Intra-day and inter-day precision and accuracy of the two analytes in rat plasma (n = 5).

| Analyte | Nominal concentration (ng/mL) | Intraday (n=5) | | | Inter-day (n=5) | | |
| --- | --- | --- | --- | --- | --- | --- | --- |
|  |  | Mean ± SD | RSD% | RE% | Mean ± SD | RSD% | RE% |
|  | 10 | 10.06±0.16 | 1.40 | 0.60 | 10.04±0.13 | 0.87 | 0.40 |
| Tacrolimus | 100 | 101.11±1.53 | 0.92 | 1.11 | 100.30±2.75 | 2.08 | 0.30 |
|  | 1000 | 1022.79±38.75 | 2.66 | 2.28 | 1017.63±6.90 | 0.48 | 1.76 |
|  | 10 | 10.27±0.39 | 3.20 | 2.70 | 10.13±0.17 | 1.49 | 1.30 |
| Deoxyschizandrin | 100 | 102.30±3.21 | 2.31 | 2.30 | 101.45±2.01 | 1.50 | 1.45 |
|  | 1000 | 1013.99±25.44 | 1.89 | 1.40 | 1011.47±18.43 | 1.22 | 1.15 |

Table S3: Stability of two analytes in different conditions (n=5).

| Analyte | Nominal Conc.  (ng/mL) | Bench-top stability  (8h, RT) | | Auto-sampler stability  (24h, 4℃) | | Long-term stability  (1 month, -80℃) | | Frozen-thaw stability  (3 times) | |
| --- | --- | --- | --- | --- | --- | --- | --- | --- | --- |
|  |  | Mean ± SD | RSD% | Mean ± SD | RSD% | Mean ± SD | RSD% | Mean ± SD | RSD% |
|  | 10 | 10.39±0.41 | 3.95 | 10.23±0.51 | 4.89 | 10.35±0.14 | 1.35 | 10.55±0.54 | 5.21 |
| Tacrolimus | 100 | 102.33±1.53 | 1.50 | 103.90±2.74 | 2.64 | 101.09±1.01 | 1.00 | 110.23±5.21 | 4.73 |
|  | 1000 | 1009.93±11.80 | 1.17 | 1010.05±13.85 | 1.37 | 1008.72±11.30 | 1.12 | 1025.45±12.50 | 1.22 |
|  | 10 | 10.63±0.83 | 7.81 | 10.23±0.93 | 9.10 | 10.37±0.25 | 2.41 | 10.45±1.01 | 9.66 |
| Deoxyschizandrin | 100 | 102.39±2.82 | 2.75 | 104.49±5.32 | 5.09 | 101.86±11.38 | 11.17 | 111.56±12.48 | 11.12 |
|  | 1000 | 1014.96±11.94 | 1.32 | 1024.65±13.44 | 1.31 | 1012.51±45.24 | 4.47 | 1050.50±50.54 | 4.81 |
